# Supplementary material for: Patterns of Intron Gain and Loss in Fungi
Source: PLoS Biol. 2004 Nov 30;2(12):e422. doi: 10.1371/journal.pbio.0020422 (PMC532390; doi:10.1371/journal.pbio.0020422)
Supplement: Table S1 — Also available at http://genes.mit.edu/NielsenEtAl/. (4.3 MB ZIP). [file pbio.0020422.st001.zip › NielsenEtAl/html/1320.html]

AN6006.1.NCU01939.1.MG02927.1.FG08910.1


```
 CLUSTAL W (1.82) Multiple Sequence Alignments - Introns Inserted


Sequence 1: NCU01939.1	1047 aa
Sequence 2: FG08910.1	1066 aa
Sequence 3: MG02927.1	1053 aa
Sequence 4: AN6006.1	1048 aa
Alignment Length: 1074 aa
Number Identitical Residues: 519 aa
Alignment Score (without introns) 26179


MG02927.1 	MDAAAVRNCIVATLDPTADVRKQGEHELKQ0AEQQPGFIDCLVTILQSEQQQNVRLGT1V
NCU01939.1	MDANAIRSCIVATLDADADVRRRAELQLKQ0AEQQVGFTDVLLDLLQTEQDVNLKLST1V
FG08910.1 	MDTAAVRSLLATSLDPDADSRRRAEIQLKQ0IEEQPGFLECLLDILQAEQEASVRLST1V
AN6006.1  	MDVAALRNRIQSTLDANADIRRQAELDLKY0AETQPGFINALLDILQGEQVNAVQLSA1G
          	**. *:*. : ::**. ** *::.* :**   * * ** : *: :** **   ::*.:  

MG02927.1 	IYLKNRVHRGWSDGESAS---SEPAINEDEKTRFKENLLPLLASSQGVIRQNLIPILQRI
NCU01939.1	IYLKNRVNRAWQRSDDY---PQEAVLDEDAKARFRDRLLPILASSETLVRHQLVPILQRV
FG08910.1 	IYVKNRVNRSWYNNEGYSTEPPSNPIPEDEKARVRDRLLPILAASEGLVRQQLIPVLQRI
AN6006.1  	VYLKNRINRGWSTIEDSP---LRAPIAEEEKPGFRERLIPALVSTPPNVRAQLVPLLQKI
          	:*:***::*.*   :. .       : *: *. .::.*:* *.::   :* :*:*:**::

MG02927.1 	LHWDFPQKWPAFMDYTVELLGTNDKDRVLAGLQCLLAICRAYRFKPNDDENKAQLQKIIE
NCU01939.1	LHHDFPEKWPTFMDYTVQLLNTNNAPSVLAGLQCLLAICRSFRYKMSDTDHRAQFEKIVE
FG08910.1 	LQYDFPARWPRFMDFTLELLNTNNPGSVLAGLQCLLAICRAFRYKSTDSDDRQHFDQIVE
AN6006.1  	LQHDFPEQWPGFLDITMQLLGTNDAGSVYAGLQCLLAICRVYRFKAGD--KREEFDKIIE
          	*: *** :** *:* *::**.**:   * *********** :*:*  *  .: .:::*:*

MG02927.1 	GSFPRLLDICRELVVQESDDAGEMLHIALKAFKHATF0LELHGILLQQETNMAWCDIFLR
NCU01939.1	VSFPRLLAVCDELVNQDSEEAGEMLHIALKAYKHTAW0LELPACLREQSVNLGWCTIFLR
FG08910.1 	ASFPRLLAICNELVNQESDEAGEMLHLALKAYKHATW0LELSPTLRQRDVNIAWCTVFLH
AN6006.1  	HSFPQLLSIGSRLVDEESVEAAEMLRIVVKSYKHAIY0FELSPHLQSHQATVDWCTLFLR
          	 ***:** :  .** ::* :*.***::.:*::**: : :**   * .:...: ** :**:

MG02927.1 	TVSRAVPASAMADESPEREKHHWWKAKKWGYYNLNRLYQR2HGNPE--AFPKDATAQLDF
NCU01939.1	TVSKPIPVSAMQDDPLGRERHHWWKAKKWAYFNLNRLYIR2YGNPHNIMEKGADGQLLQF
FG08910.1 	TVSKACPASAMQGDQHEREKHHWWKAKKWAFFNLNRLFIR2HGNP---ASPGKGEEALAF
AN6006.1  	IIAKEPPANSMMESKEERELAHWWKCKKWAYANLNRLFIR2YGNPTT-IPKSSTPDYSQF
          	 :::  *..:*  .   **  ****.***.: *****: * :*** .            *

MG02927.1 	AKYFSSTVAPAILKHYLVEIEKWVAKTTWLSRPCLSYIIVFLDECIKPKEMWTHLKPDLN
NCU01939.1	AKNFIAQVAPEILKHYLAEIEKWVAKTVWLSRPCLSYTLVFLDECIRPKEMWTHLKPHLT
FG08910.1 	AKDFTANIAPEILKHYLQEIEKWVAKTSWLSRPCLSYTLVFLDESVRPKEMWAHLKPHLT
AN6006.1  	AKSFITTFAPEILKGYLQEIDKYVSKGQWLSNPALSYTLIFFEECVKPKSMWDHLKPHME
          	** * : .** *** ** **:*:*:*  ***.*.*** ::*::*.::**.** ****.: 

MG02927.1 	NLIQHFIFPVMCLSEDDVNDFEDQPEEYLHRKLNFYEEVSAPDVSATNFLVNLTKSRRKQ
NCU01939.1	NLVTHFIFPVLCLSEEDVENFEEEPEEYLHRKLNFFEEVSAPDVAAVNFLVSLTKARRKQ
FG08910.1 	NLVTHFVFPVLCLTEEDVEQFEDEPDEYLHRKLNYFEEASAPDVAATNFLVNLTKNRRKE
AN6006.1  	NLIAHFVFPILCQSDEDIELFETDPSEYLHRKLNYYEEVSAPDVAATNFLITLTKNRKKQ
          	**: **:**::* :::*:: ** :*.********::**.*****:*.***:.*** *:*:

MG02927.1 	TFEVLQFINNVVNEYEAAAPESKNHIAKEGALRMIGTLAPVLLSKKSPIADQVEYFLVRY
NCU01939.1	TFEILKFVNEVVNQYEQAPEDQKNHLAKEGALRMIGTLAPVILGKKSPIADQVEYFLVRY
FG08910.1 	TFEILKFVNAVVNEYEQVEDDKKNHIAKEGALRMIATLAPVILGKKSPIADQVEYFLVRY
AN6006.1  	TFSILTFVNGIVSKYEAAPDDQKLPREKEGALRMIGSLASVILGKKSPIADQVEYFFVRH
          	**.:* *:* :*.:** .  :.*    ********.:**.*:*.************:**:

MG02927.1 	VFPDFTSTQGVLRARACDTIEKFEALDFKDQNNLLTIYRNILQCMADPDLPVRVTAALAL
NCU01939.1	VFPDFTNPQGFLRARACDTIEKFEQLDFKDQQNLLTVYRHILDCMADPKLPVRVTAALAL
FG08910.1 	VFPDFTSTQGYLRARACDTIEKFEQLNFQDQNNLLTIYRHILDCMADEALPVRVTAALAL
AN6006.1  	VFPEFRSPHGFLRARACDTLEKFEQLDFQDPNNLMIIYRNILESMTDSELPVRVEAALAL
          	***:* ..:* ********:**** *:*:* :**: :**:**:.*:*  ***** *****

MG02927.1 	QPLIRHDIIRRSMQESIPTIMQQLLKLANEADIDALANVMEDFVEVFAKELTPFAVALSQ
NCU01939.1	QPMIRHEIIRTSMQQNIPTVMQQLLKLANEADIDALANVMEDFVEVFATELTPFAVALSE
FG08910.1 	QPLIRHDIIRTSMQQNIPTIMQQLLKLANEADIDALANVMEDFVEVFATELTPFAVALSE
AN6006.1  	QPLIRHDVIRTSMQQNIPQIMQQLLKLANEVDVDALANVMEDFVEVFSAELTPFAVALSE
          	**:***::** ***:.** :**********.*:**************: **********:

MG02927.1 	QLSHTYLRIVRELLDKNGGRDG-EDDYADFLDDKSITALGVLQTIGTLILTLESTPEVLQ
NCU01939.1	QLRDTYLRIVRELLENNERRDGAEDEFGDYLDDKSITALGVLQTIGTLILTLESTPEVLL
FG08910.1 	QLRDTYMRIVRELLEK-ESKVGDDGELYNEYDDKSITALGVLQTIGTLILTLESTPDVLL
AN6006.1  	QLRDTYMRIVGELLER-NAAKGEEDTYGDFLDDKSITALGVLQTIGTLILTLESTPDVLL
          	** .**:*** ***:.     * :.   :  *************************:** 

MG02927.1 	AIETVLMPVIEITLENKLYD1LYNEVFEIIDSCTFAAKNISPIMWKAFELIHQTFKSGAE
NCU01939.1	HIESVLMPVIQITLENKLYD~LYNEVFEIIDSCTFAAKQISPNMWQAFELIHTTFKSGAE
FG08910.1 	HIEAVLMPVIKVTLENKLYD~LYNEVFEIIDSCTFAAKSISPTMWQAFELIHTTFKAGAE
AN6006.1  	HLETVLMPVISITLENKLYD~LYNEVFEIIDSCTFASKSISPTMWQAFELIHKTFKAGAE
          	 :*:******.:******** ***************:*.*** **:****** ***:***

MG02927.1 	LYLEDMLPALDNYVQYGAPHLVQNPQYSHALFDMVKTMFEEPKVGNVDRICACKLGEAMM
NCU01939.1	LYLEDMLPALDNFVQYGAPQLVQKPEYIQALFEMVQDMFNDTKVGGCDRICACKLSEAMM
FG08910.1 	YYLEDMLPALDNFVQFGAPQLAQKPEYTQALFSMVADMFTDSIQGGVERICACKLAEAMM
AN6006.1  	LYLEDMLPALDNYVAYGSQTLVQNPAYLAAIVGMVEDIFNDEKVGGVDRICGCKLAETVM
          	 ***********:* :*:  *.*:* *  *:. **  :* :   *. :***.***.*::*

MG02927.1 	LSLRGSIDNYIEGFVTMAMNIITSQDIKIKSVKIHLMEMVINAIYYNPLLTLQVLESNQW
NCU01939.1	LSLRGHIDQCVQGFIGLAMNVLTSQDVKVKSYKIHLMEMVINAVYYNPLLTLQILESQGW
FG08910.1 	LSLKGQIDSCVEGFINIAMTILANQDVKVKSYRIHLMEMVINSIHYNPLLTLQVLENKGW
AN6006.1  	LNLRGYVDQYIPVFIELAMRVIDAGEARTKSYRLHLMEMVINAIYYNPVLSLQVLEAKGW
          	*.*:* :*. :  *: :** ::   : : ** ::********:::***:*:**:** : *

MG02927.1 	TNKFFSLWFSSMDLFTRVHDKRLCIVAISALLSINPEQIPASVSTGWPRLLQGITVLFRT
NCU01939.1	TNKFFSLWFSSMDSFSRVHDKKLCIVAIVALISIPADQIPPTVAVGWPRLLQGITSLFGS
FG08910.1 	TNRFFSLWFGSMTAFTRVHDKKLCIVAISALLSLNPEHVPQSVSVGWPRLLQGITELFRS
AN6006.1  	TNKFFSAWFSSIDSFRRVHDKTLSIVAITSLLTLNPADVPTSVQQGWPRLLQGVTRLFHT
          	**:*** **.*:  * ***** *.**** :*::: . .:* :*  ********:* ** :

MG02927.1 	LPNAEKN~RAEAMKD-DFTLDPGSYDYEDDDEWDEDDANWNADGDQEE-EPSDVKDESTA
NCU01939.1	LPTALKN~REEALKD-DYHLDGG--VYDENEEWDDDENNWDAGEEGEEEDLGDVKGESEA
FG08910.1 	LPAAQKN1RDEALRD-DFHLESTY-DYGEEDEWDDDEANWNVEEEEETGETAESKDESTA
AN6006.1  	LPAAIQN1RQEATKESDFQYEEED-EDDEGNDWEG-EVEWTEG-DDEAGPEGDIPDESAA
          	** * :* * ** :::*:  :       : ::*:  : :*    : *    .:  .** *

MG02927.1 	YIEFLNEE0AQKFSNLDQGGFESDDDLGEQSLLLETPLEKVEPYGIFRTVLLR1LQQEQP
NCU01939.1	YLEFLNEE0AQKFSGTDLE--YSDDELGEDGVLLESPLDKLDPYSIFKTSLLK1LQAEQP
FG08910.1 	YLNFLNEE0AQKFSRAIDD--VEEDDLGEDSVLLESPLDKIEPYQLFRNTLMK1MQAEQP
AN6006.1  	YLDFLNKE0AAKFGSFAGD--DDDDELDEE-SLLETPLDKVEPYGLFKQVFMN1LQQQQP
          	*::***:* * **.        .:*:*.*:  ***:**:*::** :*:  ::. :* :**

MG02927.1 	QFYQSLAGHLQDDDRNMLQTVVQQAEQ-----------IMIVAQQQQQAMGQG--AANGG
NCU01939.1	QYYAGLVQHLSMEEQSVISAVLQQAD------------TQEAAAQQAAAAAN-------G
FG08910.1 	QFYGSLAGHLTADDQNVIQNVMVKADEIATQQAQQAQQAQLLAQQQAAAMAASLGAQNGG
AN6006.1  	QLYENLTNILNDEEKKIIESVFHEADA-----------KALAAANEEAAKAAA--LQANG
          	* * .*.  *  :::.::. *. :*:                * ::  * . .     .*

MG02927.1 	AS
NCU01939.1	TS
FG08910.1 	AS
AN6006.1  	NQ
          	 .
```
